# Footprint Reduction of Sensor Control Modules for Remote Portable Laboratories

**DOI:** 10.3390/s22041483

**Published:** 2022-02-14

**Authors:** Sebastian-Alexandru Arghirescu, Maria Drăgan, Octavian Fratu

**Affiliations:** 1 Campus Centre, University “Politehnica” of Bucharest, 061102 Bucharest, Romania; sarghirescu@upb.ro (S.-A.A.); maria.dragan@upb.ro (M.D.); 2 Telecommunications Department, Faculty of Electronics, Telecommunications and Information Technology, University “Politehnica” of Bucharest, 061071 Bucharest, Romania

**Keywords:** sensors, automation, robots, autonomy

## Abstract

Following the automation of monitoring systems for pollution levels in cities or protected nature reserves, there comes a need to increase the autonomy of robotic vectors deployed in the field. Thus, it is important to consider the weight that these robots must hold in order to be able to carry out a comprehensive analysis of the environment. A balance must be struck in the size, weight, and complexity of the mobile laboratories used for measurement and the autonomy of robots, especially given that current technology does not allow, in most cases, a completely autonomous battery charging cycle. Thus, in this paper, we consider a microcontroller-based architecture for a mobile laboratory control system that will be used for installation on both an aerial and an aquatic mobile vector. We found that such a system can be repurposed for several sensor types and configurations, thus being able to massively reduce the space allocated when compared to embedded widespread products.

## 1. Research Strategy

The aim of this paper was to design and analyze a smaller footprint control system for a sensor array that will be used as a mobile laboratory for a remote-controlled drone, as well as a remote-controlled boat. The purpose of this is to reduce strain on the battery and increase measurement autonomy. Currently, mobile labs are either too large [1] or unable to be repurposed enough [2] to be practical as robot attachments for multiple scenarios. Our design allows for control via a microcontroller platform (including the possibility of using an Arduino device [3]) of a sensor system from which both digital signals are acquired in positive logic 1 format (when the respective input is logic 1, the sensor is in working condition and does not detect the parameter related to that sensor, whereas logic 0 means a faulty sensor). In addition to logic state acquisition, the platform allows receiving 16-bit analog signals from multiple sensors. Additionally, the platform will be able to control, through the same software, via appropriate logic signals, different relays required by the application, as well as data display devices such as optical displays, LEDs (light-emitting diodes), and buzzers. The system will be able to collect the required information from semiconductor resistive sensors and electrochemical sensors.

There are some relevant articles and similar solutions that have surfaced during the past few years that are worth discussing further with respect to our proposed system. There is a solution [4] consisting of an Espressif Systems ESP 32 microcontroller with a Heltec Automation Lora 32. It includes a Li–Po (Lithium Polymer) battery management system and an OLED (Organic Light-Emitting Diode) screen. It uses the internal 12-bit ADC (Analog-to-Digital Converter) of the microcontroller. It only senses CO_2_ (Carbon Dioxide) emissions. The operating voltage is limited to 4.5 to 5.5 VDC (Volts of Direct Current), and the average current sunk from the control board is 60 mA maximum at 5V. This solution is more focused on back-end integration with AirSensing Mobile and Web services. It also provides BLE (Bluetooth Low Energy) connectivity for sending the data directly from the sensors to a mobile computer in order to be stored by the. NET application in an MS SQL Server database.

The authors of [5] were not focused on a hardware control module solution, but instead assessed the importance of air quality through questionnaires and physical measurements. They concluded that 80% of the responders were satisfied with the perceived indoor air quality. This is important as it gives us a reason to shift out focus toward outdoor monitoring and control systems. Our system should, therefore, be able to resist the harsh weather conditions associated with outdoor use. In [6], the authors presented an assessment of air-quality microsensors as compared with reference methods. They tested various refence air quality measurement stations and microsensor solutions such as the Cambridge University SNAQ (Sensor Networks for Air Quality) boxes for AQ (Air Quality) monitoring, deployed in Cambridge and London Airport for air-quality studies, the Aristotle University ISAG microsensor box, ECN airbox as a water-resistant unit, the NILU + Envira NanoEnvi platform, the DAEA-CSIC AQMesh node, the ENEA air-sensor box, the VITO EveryAware SensorBox, the UCL/CCMOSS MOS micro-hotplate for relative humidity sensing, the S OdorCheckerOutdoor, and the Siemens AGeGa2O3-based micro-hotplate sensor. The authors mounted these solutions on an outdoor mobile lab van. The authors concluded that these microsensors were able to measure the levels of O_3_ (Ozone), CO (Carbon Monoxide), and NO_2_ (Nitrogen Dioxide) with relative success from the tested platforms. There were also gases that were profiled well. For PM (Particulate Matter) and SO_3_ (Sulfur Trioxide) the results were shown to be poor, with low correlation coefficients between them and the reference. This study shows what sensors should be looked at for compatibility purposes with our proposed system, as traditional reference sensors are too large to be used in remote controlled mobile labs. The authors of [7] tried to determine the effectiveness of the variables influencing indoor air quality. This can be further extended to outdoor air quality, as they also reviewed typical models for determining air pollutants and their relationship with health effects. 

In [8], the “Trust V” model was presented for increasing trust in autonomous systems. Ways of increasing the trust during the engineering phase of system development were proposed, so as to not need it after the fact. Ways of measuring operational trust in systems were also presented. This is an article that should be followed in the design philosophy of any solution. It is important in the context of possible further developments of our solution as we would like to pursue automating the cooperation between the land and air mobile labs used for testing the sensor control system. The authors of [9] tried to compare different object detection technologies in terms of power cost and performance, and they also looked at the object detection evolution over the last few years. They concluded that precise object detection systems such as radars that can be used in small systems are getting increasingly cheaper, lighter, and more accurate. In addition, they presented ways in which signal processing is used to increase the performance of these sensors. In [10], the question of imposing backup plans in autonomous systems was raised, such as kill switches and human-centric remote control stemming from safety concerns. This is important to consider as new standards and regulations surface for controlling autonomous systems so we can make sure that any safety measures are implemented in the design phase and not thrown together afterward, thereby hurting overall system performance. 

In [11], the recent advancement in network control systems was presented in the form of a time-delay system approach, Markovian jumping system approach, switched system approach, stochastic system approach, and impulsive system approach. Various methods of network-based filtering were also shown, while raising some of the yet unsolved challenges such as the fact that network-induced delays and packet dropouts are explicitly dependent, and that, in event-triggered control and filtering, it is often to assume that packet dropouts and packet disorders do not occur. This is a good starting point for developing a future network communication system for mobile remote-controlled labs. The authors of [12] showed how these kinds of control systems for sensors can be used to detect and prevent wildfires and other natural disasters. The system shown there works by stopping power in various main segments depending on data coming from voltage spike sensors. This is also a possible application of our system. We can detect various gas leaks or voltage spikes and open water valves in ventilations systems or circuit breakers with the right sensors and actuators in order to prevent further harm. The authors of [13] presented solutions to control system problems caused by random sensor delay by implementing an integral sliding mode control scheme. There were numerous numerical examples and simulations presented. The work presented in [14] is useful for understanding how we can assure future compatibility with the implementation of a mobile communication module for transmitting and storing sensor data in a database without increasing latency and loss for multiple-sensor systems. 

These articles present a good starting point for developing a solution which fixes the portability problem of most sensor systems, as well as being modular enough to be used for various applications for air-quality measurement in hard-to-reach places, as well as to warn governments of air pollutant levels in busy cities through automated systems and to save lives in natural disasters. It is also important to keep the cost down in order to increase the adoption factor. The shortcomings of the existing methods are the large sizes of accurate systems and the lack of sensor compatibility and adaptability of the small designs. We aimed to come up with a middle-ground design in the sense that our system should be able to support more sensors while still being able to be adapted to numerous more specific applications. The main problem we attempted to solve with this paper is the adaptability of the design. Our solution must be able to accept a wider variety of sensors. It also must be able to adapt to power constraints of different applications while not sacrificing the portability of other solutions. 

Some possible applications that we are consider in designing the system are described. Firstly, the design should be able to be used for cheap LPG (Liquefied Petroleum Gas) gas leaks in canisters transported to and from gas stations, minimizing risks of leaks and explosions. In this case, the design should accept visual and audio warning components and accommodate and power an LPG gas sensor in addition to the power requirements of the control board. Such cheap and widely available sensors exist in the form of the Winsen MQ sensor lineup. The MQ-5 is an example of a sensor from this lineup which can measure LPG concentration in air. These sensors and their datasheets can be found in [15] and are good examples of the types of sensors that the controller board should support. Another application of these sensors could be natural gas spread detection in mines. The low cost of the system could provide the opportunity to mount numerous system samples throughout the mine and detect the spread of gas. We could use MQ-4 or MQ-7B as an example for this application. Our design should also be able to accept various actuators so that the data from these sensor boards can then be used to estimate gas movements within the mines and open various ventilation vales in accordance with the air-quality level. Other applications could be alcohol and ammonia gas detectors in labs or cheap carbon monoxide detectors in homes. As such, the design should be based on widely available parts. It is also important to be able to calibrate the sensors to be useful in these applications. The MQ line also provides a method for coarsely calibrating the sensors by adding an external resistor in order to control the gas concentration baseline with the following general formula:

(1) $$\frac{R_{s}}{R_{0}}=\frac{Gas\;concentration\;\left(\mathrm{ppm}\right)}{10^{4}},$$

where $R_{S}$ is the resistance of the sensor in target gas, and $R_{0}$ is the resistance of the sensor in clean air. This formula is then particularized for each sensor on the lineup inside each datasheet. We can, thus, say that the applications for this kind of design go beyond the need for small and light sensor modules for installation on remote controlled labs.

The remainder of this paper is organized as follows: in Section 2, we present the system description, more specifically, the parts used, block diagrams, schematics, and code descriptions. In Section 3, we organize the results and the possible system applications. In Section 4, we compare our system with other similar solutions, drawing conclusions and presenting ideas for future developments.

## 2. Research Activities and Technologies Used

**Figure 1:**
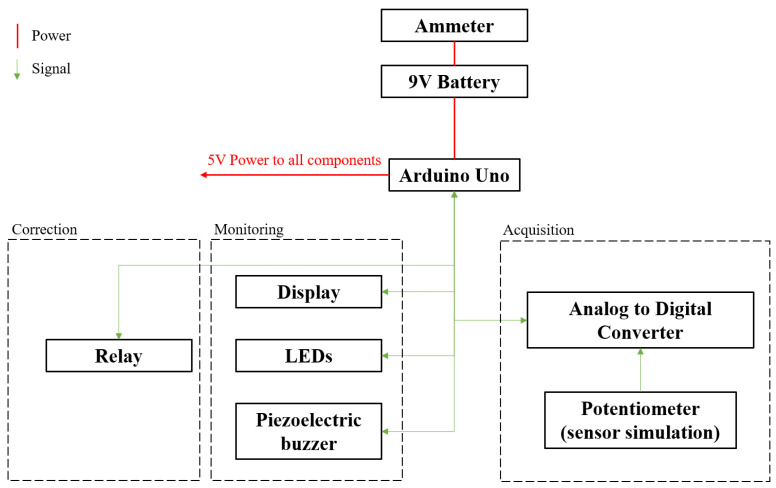
Block diagram of the proposed system.

For our experiments, we developed a system with the block diagram in Figure 1.

We decided on using an Arduino board as the basis for testing our system. This board has a ATmega328P as the microcontroller which we use for receiving the sensor data, as well as for controlling the actuators and warning LEDs. We use a 9 V battery which is stepped down to 5 V by the voltage regulator on the Arduino board for powering the system, as well as an ammeter for measuring the current consumption of the system. We use an external ADC as outlined in the table below, as the internal ADC of the 328P only stores 8 bits of data per sample. We use a piezoelectric buzzer, as well as a green and red LED, for gauging the level of perceived simulated air quality. We also use a relay to act as an actuator which opens if there is a warning state detected. The display also shows this warning signal when the simulated air pollution goes over an arbitrarily set threshold. For simulating data from any air-quality monitoring sensor, we use a potentiometer on the input of the external ADC. In short, we acquire the signals from the sensor using the ADC, we monitor the system using LEDs, a buzzer, and a display, and we correct problems using a relay.

We used the equipment listed in Table 1.

It is worth mentioning with regard to the requirements listed above that an Arduino development board cannot sample at 16 bits. The vast majority of their products sample at 10 bits, with the exception of Zero, Due, MKR, and Nano 33 (BLE and IoT) cards which can use a special *analogRealResolution()* function [23] in order to increase the sampling resolution to 12 bits. There is another limitation of the Arduino internal ADC (analog to digital converter), whereby the default sampling frequency is 9.6 kHz. In this case, it can only sample signals up to

(2) $$F_{Nyquist}=\frac{9600}{2}=4800\;\mathrm{Hz}.$$

Furthermore, the microcontroller can sample at a resolution of 10 bits only up to a maximum frequency of 15 kHz [24], after which the resolution decreases to 8 bits. The maximum possible sampling frequency by changing register values at the microcontroller level is 76.9 kHz.

Consequently, we would require an external ADC that can sample at 16 bits. We should make the choice depending on the application. In this case, we are using resistive and electrochemical sensors; thus, the resolution dramatically precedes the sampling frequency. We chose the ADS1115 module. It can sample at a resolution of 16 bits (or 15 unsigned), uses an MSOP (Mini Small Outline Package) capsule, thereby not taking up much space, is easy to work with, and can be powered by 5 V directly from the microcontroller board voltage regulator.

Using this module, we designed a test system according to the block diagram in Figure 1 which can simulate the operation of any resistive sensor in terms of output sampling using a potentiometer.

**Figure 2:**
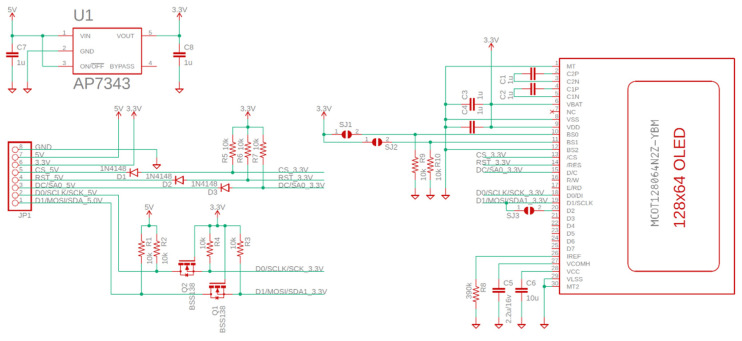
Schematic diagram for the OLED screen.

This potentiometer is placed with the outermost pins between the power supply (5 V) and ground, and the variable terminal is connected to the Arduino A0 pin. Thus, we have a resistive divider that creates a voltage point varying between 0 V and 5 V (approximately). We use this potential to simulate the output of a sensor, and we can manually change the value for testing. In the diagram, there is also a green LED and a red one, which show us if the value of the sensor is lower (red) or higher (green) than half of the whole dynamic range (5 V). These LEDs are connected to Arduino digital pins via 270 Ω current-limiting resistors, thus limiting the current to 10 mA. The OLED screen board was designed by us in the past and is based on a panel sold by Adafruit [25]. It can be controlled by SPI-3 Wire, SPI-2 Wire, or $I^{2}C$. The wiring diagram of the OLED control board can be found in Figure 2. The OLED displays whether the signal value is below (“Warning !!!”) or over half (“Value OK”) of the dynamic range. We also introduced a buzzer on the board along with a relay, which becomes active when the signal stays below half the dynamic range, approximately 2.5 V in this case.

As part of the design presented in Figure 2, we included a low-power linear voltage regulator (U1) as the OLED controller runs on 3.3 V, and the rest of the board is powered from 5 V. In this sense, we also included bidirectional logic level translators in the form of Q1-R3 and Q2-R4, as well as unidirectional translators in the form of D1, D2, and D3 with their respective pullup resistors. The other components are required from the datasheet of the SSD1306 OLED controller and are used to filter noise and set the SPI address for the controller.

The code description can be found in Algorithms 1 and 2, together with references to the additional libraries used. 

We start the code by including some necessary libraries in order to control the OLED screen, as well as the SPI communication between the external ADC and the microcontroller. The libraries in question are *SPI.h*, *Adafruit_GFX.h*, *Adafruit_SSD1306.h*, and *Adafruit_ADS1X15.h*. We then appropriately initialize all of the hardware modules and start the setup function. The *Setup* function is only run once upon power-up of the system. Inside this function, we catch possible errors relating to improper initialization of either the ADC or the display, as well as write the registers for various microcontroller pins according to their functions such as setting pins 5, 6, 7, and 8 as outputs in order to let the microcontroller take control of the status LEDs, relay, and buzzer. We also wait 20 ms. This value is specified in the OLED datasheet and is required for proper initialization. A lower value of 10 ms was also shown to work but it could lead to instability over prolonged use. 

Both the *Setup* function presented above, and the main loop function should not take any external variables. The *Loop* function reads all of the sensor variables from the ADC, stores them in *ADC_Val* at every iteration, and converts these raw values to voltages (fractions of the main bus voltage 5 V stored in *ADC_Volts*) for easier processing further in the code, as well as for easier debugging in the serial monitor. Using these values, we can now use warning LEDs to signal dangerous pollutant levels in the air, we can actuate any electromechanical devices in order to correct the air quality (relays, valves, ventilations systems), or we can send this data to any cloud database. In the context of this code, we turn on a red LED connected to pin 8 and a piezoelectrical buzzer on pin 6, as well as open a relay on pin 5, if the measured pollutant level from the sensor is above a certain test threshold deemed dangerous. We turn on a green LED on pin 7 and turn off the relay and the buzzer if the measured level falls below the threshold. This can be extended to any application with a large number of small sensors (with a total draw of no more than 2A). The microcontroller used here (the ATmega328P) can also be upgraded to one with more general purpose I/O pins for even more sensors and/or actuators if the application demands it.


**Algorithm 1: Initialization**

**Including SPI library <SPI.h> found at [26]**

**Including OLED display library <Adafruit_GFX.h> found at [27]**

**Including OLED control library <Adafruit_SSD1306.h> found at [28]**

**Including external ADC control library <Adafruit_ADS1X15.h> found at [29]**


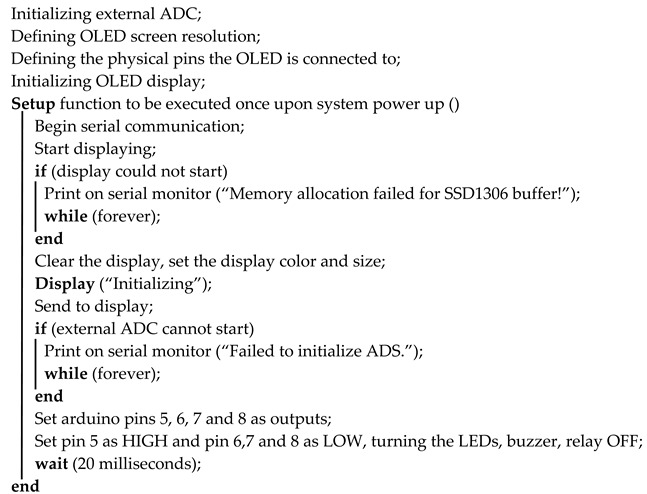



**Figure 3:**
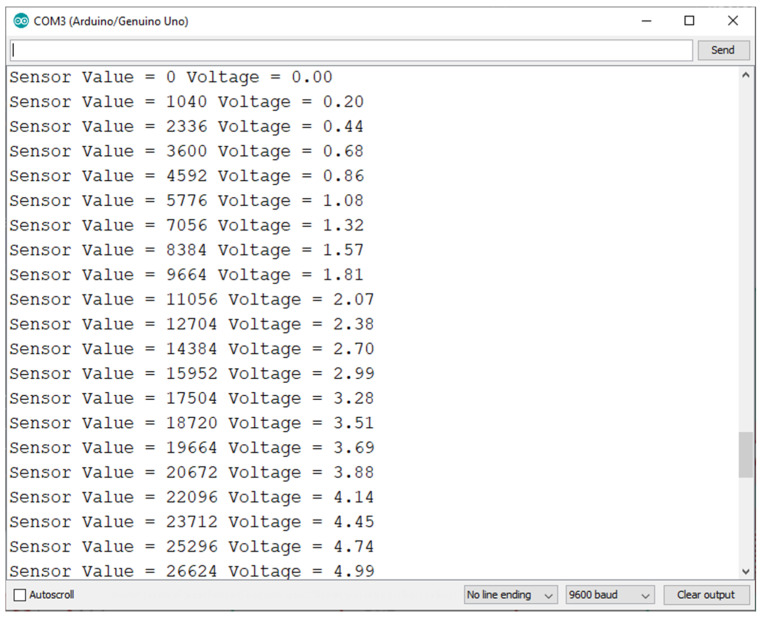
Serial monitor output for various detected input voltages.

The code also includes various test instructions in the form of *Serial.print* in order to display the sampled value at the current time together with the corresponding voltage in volts on the serial monitor, as we can see in Figure 3. It shows the interface of the serial monitor we used for debugging the code, including fine-tuning the ADC value to voltage conversion. We can see that, if we vary the test sensor input, which would translate to the level of air pollutants increasing around the sensor, the value at the output stays between 0 and 5 V, covering the entire dynamic range of the ADC. In our specific case, a value of under 2.5 V would mean that the level of pollutants read by the sensor is under an acceptable threshold (the green LED would be on), and a value of over 2.5 V was set to trigger the buzzer, relay, and the red warning LED. In our test, we sample at a rate of 860 samples/s. Only some of those samples are sent to the serial monitor for visual review. We can, thus, see that the conversion between the ADC output values and the voltage values is working as intended.


**Algorithm 2: Reading sensor data, actuating relays, and warning LEDs**


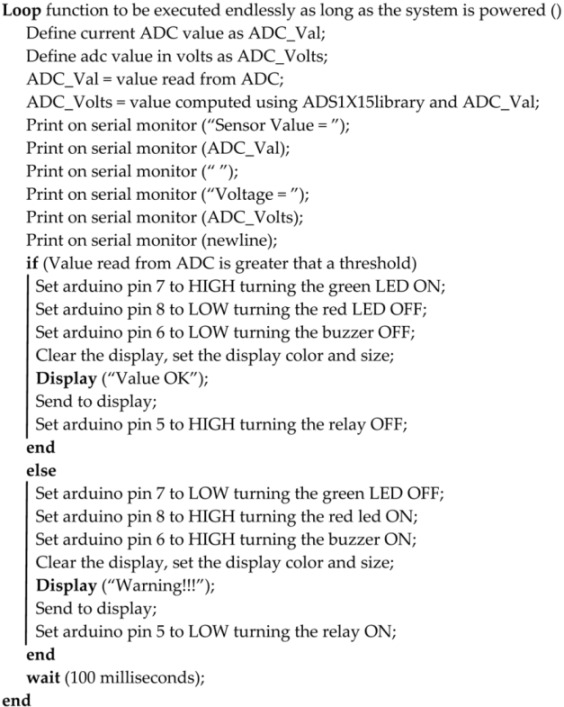



Figures depicting the operation of the test system, together with its detailed wiring diagram, can be found below.

**Figure 4:**
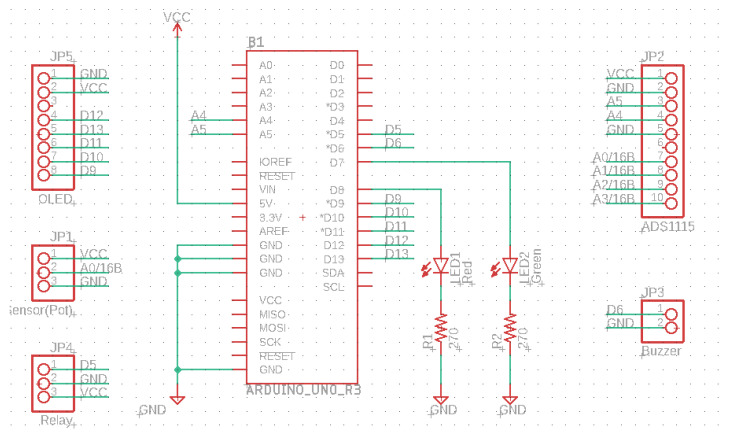
Connection diagram for the system.

Figure 2 and Figure 4 show the schematic diagrams of the system and the OLED control board. It is important to note that the schematics use net aliases for unconnected nets. The pin headers on Figure 4 represent the various sensors used.

## 3. Research Details and Results

For simulating a possible use of the system design, we developed a test scenario. The components were interconnected as shown in Figure 1. We set up the system in such a way that it could detect when a pollutant threshold was reached (and subsequently passed). This threshold was set at half of the full detecting range of the simulated pollutant sensor (the potentiometer in Figure 1). The idea of this test is to check if we could safely and remotely regulate the air-quality level surrounding the system by issuing a warning and activating an actuator if the system detected a pollutant level over the set threshold. In the case of this test scenario, the actuator does not regulate the potentiometer value after it activates; we purely tested whether it would activate and deactivate in time.

**Figure 5:**
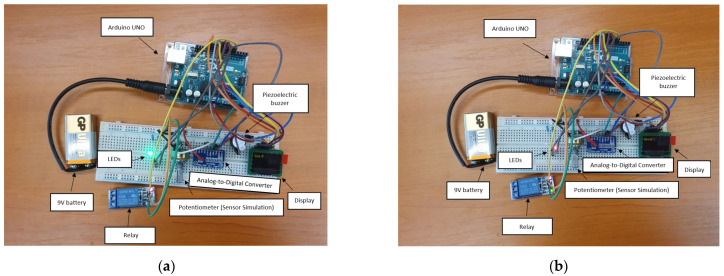
Prototype of the system used for testing, with simulated sensor value over half of full scale (**a**), and with simulated sensor value below half of full scale (**b**).

Figure 5a,b show the functioning principle for the prototype. When the sensor (the potentiometer in this case) returns a value over half of full scale (represented by 5 V in this case), the green LED is on, and the relay stays closed. Otherwise, the LED turns red to signify an error, and the relay opens. This system can be expanded to any number of relays and sensors.

**Figure 6:**
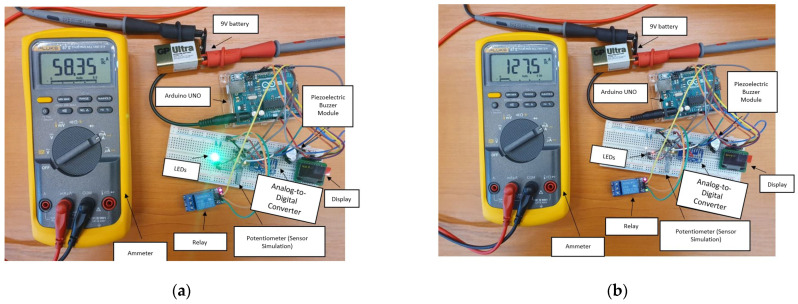
Current consumption with the relay turned off (**a**) and on (**b**).

Figure 6a,b show the power consumption of the system. We see a value of 58.35 mA with the relay and the buzzer in the off state and a value of 127.5 mA when the relay and the buzzer are both tuned on. Here, we wanted to simulate the mean current consumption when the system is simply monitoring the sensors, as well as when the system detects a warning and executes a subroutine in order to notify the user and correct the problem. The values noted above are expected to vary drastically with the number of sensors and relays. In order to mitigate some of the current consumption, especially in case where we want to mount the system to a battery-powered drone or boat, we could mitigate some of the current consumption by using small solar panels in series–parallel configurations such as the one found in [30]. As outlined in the application note of this solar panel in [31], the formula for current and voltage generated is

(3) $$I=I_{L}-I_{0}\left(e^{\left(\frac{V}{V_{t}}\right)}-1\right),$$

where *I* and *V* are the total current and voltage generated by the cell, $I_{L}\;$ is the actual current generated by the solar irradiance on the cell, $I_{0}$ is the leakage current. and $V_{t}$ represents the thermal voltage geerated due to the heating of the cell. These cells can provide 120 mA at 2 V each; hence, we would cover most of the current consumption for the test system with just three cells in series totaling 6 V at 120 mA. We could then add more groups of three cells in parallel, thus increasing the current output by 120 mA for each group. This would of course increase the cost of the system because we would require a charging control circuit for the specific battery chemistry used.

Considering the large size of the prototype, it is not exactly ideal for attachment to a mobile vector is this form. Moreover, the current consumption is relatively high as seen in Figure 6. A consumption of 60 mA in normal operation together with almost 130 mA when the relay and the buzzer are open approaches the power delivery limits of the Arduino platform. It should be noted that this consumption includes a sensor that consumes only very little current (0.1 mA). A group of real sensors can increase the total consumption up to over 500 mA, in which case it is no longer possible to use the linear voltage regulator on the Arduino board or a USB power cord coming from a computer.

In order to test the sensor system, we built a remote-controlled drone, as well as a boat. The main issue we had in mind here was adaptability and ease of modifying the layouts. 

**Figure 7:**
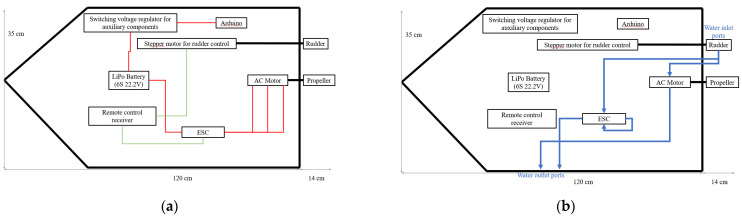
The boat electrical connection diagram (**a**) and cooling system diagram (**b**).

As we can see in Figure 7a, the boat was equipped with an Arduino module, as well as a variable switching regulator, to provide power and controllability for any modules that could be placed later, such as various sensors or communication modules. This stabilizer together with the Arduino board can be powered from the Li–Po (lithium–polymer) battery charging port. This port provides access to the individual voltages of the battery cells, although, to avoid asymmetrical discharge, we recommend using the full voltage of 22.6 V for everything. The stabilizer allows an input voltage between 3 and 32 V and can supply an output voltage between 5 and 35 V. It can provide up to 4 A of output current with an average efficiency of 94%. The output ripple was measured at 50 mV. The diagram shows power connections in red, signal connections in green, and mechanical connections in black.

The adhesive used to mount the components to the hull of the boat was based on silicone, having an activation temperature of 195 °C and retaining its full adhesion force up to 150 °C. In this case, the components can be moved if necessary but will not move under normal use cases. We also kept in mind that the bottom of the boat’s hull can accumulate a small amount of water; hence, water-sensitive components should not be placed in this area.

Water cooling allows for a much longer runtime of the engine and of the engine’s ESC (electronic speed controller) at maximum capacity, with the only limiting factor for the boat’s autonomy in this case being the battery capacity.

The drone was based on a Pixhawk 4 control module along with the power distribution board that came with this module. This allowed us to use QGroundControl to upload mission control data to the drone. 

The guide we used for configuring the drone settings can be found in the Pixhawk documentation [32].

**Figure 8:**
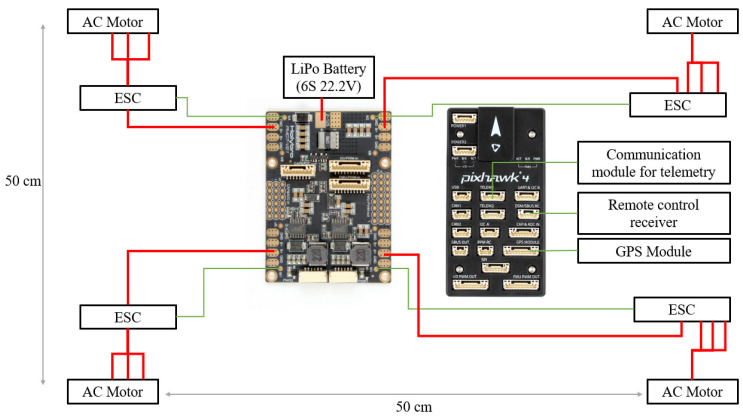
Diagram of the electrical connections of the drone.

Figure 8 shows the power connections for the drone circuits marked in red and the signal connections marked in green. It should also be noted that, for the sake of readability, some connections are missing from the diagram, namely, the POWER1, POWER2, I/O PWM OUT–IN, and FMU PWM OUT–IN ports between the control board (center right) and the power distribution board (center left). The entire circuit is powered by the same battery used in the boat, and the ESCs can be controlled manually using a remote-control receiver. The GPS (global positioning system) module is mandatory if we want to upload data for an autonomous mission, without the necessity of human supervision.

In terms of current consumption, the tests were performed in laboratory conditions, both for the drone and for the boat at a constant engine speed of 80% (all four engines simultaneously in the case of the drone) with a Li–Po 6S battery of 4.2 V per cell with a capacity of 10 Ah. The power consumption was approximately 20 A for the drone and about 5 A for the boat. This consumption led to an autonomy of about 30 min for the drone and 2 h in the case of the boat, according to the following formula:

(4) $$Autonomy\;\left(h\right)=\frac{Battery\;capacity\;\left(\mathrm{Ah}\right)}{Current\;consumption\;\left(h\right)}.$$

**Figure 9:**
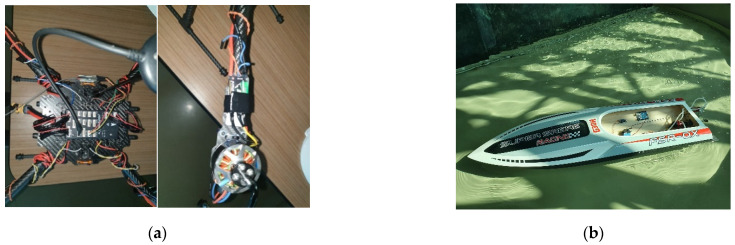
Pictures of the drone (**a**) and of the boat (**b**).

It should be noted that the consumption calculated above is expected to increase when operating in real-world conditions, and it is expected that it will also depend on the weight distribution in the case of the boat. Therefore, we recommend placing the battery in the named slot from Figure 7 after charging is complete. In Figure 9a,b, we can see pictures of the final designs for the drone and the boat.

Using the control system presented above in conjunction with these mobile remote-controlled labs, we can measure air quality in hard-to-reach places. This makes it possible to measure pollution in harsh environments where sensor boxes would require periodic maintenance and where repair personnel cannot reach. Furthermore, we could use these systems to measure air quality in places where it is important not to disturb the local flora and fauna such as natural reservations. In addition to this, the system can be used as a traditional fixed lab for air-quality measurements if a weather-resistant box is designed to fit the board. The main disadvantage here would be the loss of precision compared to larger solutions. We delve into a more detailed analysis and comparison to other solutions in the next section. 

To summarize our contribution following the results, we designed the system and the software in Section 2 in such a way to allow for implementation in various PCB designs, tailored to the designer’s needs. In this sense, we used an Arduino as the basis for our test, but the design can be adapted to any dimensions, power requirements, and sensor type needs, as long as the sensors provide an analog or board supply voltage-level signal as an output.

## 4. Conclusions and Future Recommendations

Following the automation of air-quality monitoring systems, there will always be a need to increase the autonomy of robotic vectors deployed in the field moving forward. Thus, it is important to consider reducing the weight and footprint of the electronic control and measurement systems they must carry. We must be very careful when striking a balance in the size, the weight, and the complexity of the mobile laboratories, as well as the autonomy of the robots. In this paper, we managed to design a small, adaptable control system which can be interfaced with numerous sensors and actuators. 

The solution presented in [4] is the closest implementation to our system prototype, but it still lacks in terms of future developments for space optimization. It uses an unwieldy board for the sensor module which, depending on the specific application, can prove to be too big for installation. It also uses a commercially available development board, which means it is using components which are not needed for sensor integration, further wasting available space. In contrast, our prototype can be laid out on a PCB in order to be more flexible in terms of sensor support and microcontroller support. It can be used to measure any air-quality parameters. In addition, the wires can be traced directly on the PCB, thus improving the noise rejection characteristics, which is particularly important when working with air-quality sensors with low-voltage-level signals. The working temperature and humidity would be identical between the two solutions at 0–50 °C and 0–95% respectively, but our solution would allow for a weather-resistant case to be designed around the board. Our prototype is also more flexible in terms of power delivery, being able to be powered for sources exceeding 5 V, and it can provide 1–3 A of current to various sensors compared to 60 mA. Our solution lacks BLE connectivity, but it is compatible with any Bluetooth modules that could be added in the future. The systems presented in [6] are set to be installed on big mobile labs in the form of meteorological vans and are, therefore, too big to be considered for our system, but the article shines some light on the accuracy of the micro sensors that can be installed on small remote-controlled mobile labs when comparted with their big, reference counterparts. In conclusion, considering the state of the art, we can say that the strengths of the system lie in the fact that it is far more portable than any fixed lab which is not constrained to measuring a small part of the air pollutants. In addition, this system is far more flexible than the portable lab presented in [4]. It is able to accept a wider variety of sensors, as well as a wider variety of enclosures. The main board is not constrained to using a specific microcontroller. It can be adapted to the application demands. On the other hand, the board was designed with small sensors in mind, which, considering the analysis done in [6], can be far less accurate than traditional big air-quality monitoring fixed labs. In its cheapest form, it is also not very robust, and the price will increase proportionally to the application operating conditions. In order to circumvent the first drawback, our system could perhaps be used in conjunction with a more expensive, larger, more accurate system for critical applications where portability is not a concern, thus adding redundancy to the larger system. There are also concerns tied to the compatibility of some sensors with the proposed system. It accepts all the most commonly found small air-quality sensors, but there are some requirements to be followed. The sensors should send an analog or digital signal that fits within the full dynamic range of the ADC which, in most cases, would be the supply rails of the system. Therefore, there may be some problems with the compatibility of sensors that have proprietary power adapters or that require AC power in order to function.

In future, having found the results discussed above, we propose designing a custom layout for an integrated board compatible with the Arduino development environment on which we can place additional components. As mentioned throughout the paper, this board should provide the basis for adapting other designs for specific applications. This board could then be field-tested in numerous applications such as those outlined in Section 1: natural gas distribution measurement and correction in mines, low-cost LPG gas leak detection, or carbon monoxide/ammonia leaks in research labs. The board can follow the design outlined in Figure 2 and Figure 4, keeping in mind some recommendations. Firstly, considering the size of the prototype, it is not exactly ideal for attaching to a mobile vector or other portable applications such as attaching to a bike in order to measure big city pollution levels. As such, considering the number of components required in accordance with Table 1, the board can easily be laid out on a 100 × 100 mm single-sided PCB. We recommend using a one-sided PCB for thermal constraints, especially if it has to power a large number of sensors. The copper backplane should provide adequate cooling if correctly sized in this case, without the need for bulky heatsinks for the voltage stabilizers. Secondly, the current consumption is relatively high, as seen in Figure 6. A consumption of 60 mA in normal operation together with almost 130 mA when opening the relay and the buzzer is close to the limits of the Arduino platform. It should be noted that this consumption was tested for a simulated sensor that consumes very little current (0.1 mA). A group of real sensors can increase the total consumption to over 500 mA, in which case it is no longer recommended to use the linear converter on the Arduino, and the use of USB power becomes impossible. Buck-Boost converters can provide considerably more current to the sensors, and such a power delivery system can also be designed with a smaller footprint in mind on a custom layout. Some possible designs for small-footprint Buck converters can be found in [33], supporting currents of up to 15A on a 50 × 30 mm board. Following these recommendations, we can reduce the system footprint even further by eliminating unnecessary Arduino components, such as unused GPIO (general-purpose input/output) pins and by integrating the analog-to-digital converter, LEDs, buzzer, and board relays into the PCB.

## Figures and Tables

**Table 1:** Equipment used and main parameters.

Equipment Name	Main Parameters	External References
External ADC	ADS1115 16 bit	[16]
Arduino Uno	Rev3 with 5 V signals	[17]
OLED Display	Chip-On-Tab 128 × 64 OLED with SSD1306 controller and custom PCB	[18]
Potentiometer	50 kΩ	[19]
Piezoelectric Buzzer	Active, 5 V	[20]
Resistors	2 × 270 Ω	[21]
LEDs	20 mA Green, 20 mA Red	[22]

## Data Availability

Not applicable.
